# Sofosbuvir Has Come Out of the Magic Box

**DOI:** 10.5812/hepatmon.16916

**Published:** 2013-12-16

**Authors:** Seyed Moayed Alavian

**Affiliations:** 1 Baqiyatallah Research Center for Gastroenterology and Liver Diseases, Baqiyatallh University of Medical Sciences, Tehran, IR Iran; 2 Middle East Liver Diseases Center (MELD), Tehran, IR Iran

**Keywords:** Hepatitis C, Treatment, Sofosbuvir

After the introduction of hepatitis B (HBV) vaccination, which leaded to a dramatically decrease of HBV prevalence in the communities (1), it is predicted that hepatitis C virus (HCV) will emerge as the most common chronic viral liver disease in the next decades (2, 3). The global prevalence of HCV is at least 3% with over 170 million infected cases (4). The treatment regimens for chronic hepatitis C (CHC) have progressed within the past decade. The current standard of care in our country consisting of pegylated interferon alpha (PegIFNα) and ribavirin (RBV), has significantly improved the sustained virological response (SVR) rates from 50% up to 80% in patients infected with different HCV genotypes (5, 6). Iranian patients respond well to antiviral therapies which may be related to more favorable distribution of IL28B SNP polymorphism (7). After the introduction of Boceprevir and Teleprevir as the standard therapy for HCV genotype 1 infections, the hope for eradication of infection grows significantly, but the costs imposed by the drug side effects were not satisfactory for both patients and scientists (8). Availability of these agents, protease inhibitors (PIs), which have been added to the current standard of care (SOC), has offered a new treatment option for patients infected with HCV, who are infected with GT1, and have not responded or relapsed to the previous therapies. It is reported that by adding these agents to the standard of care of patients who were infected with HCV genotype 1, non-responders or relapsers to the previous therapies, the response rate had increased. The therapy failure in patients infected with different HCV genotypes in Iran, depends on the liver fibrosis status, age and comorbidity (8). Chronic HCV infection and its treatment are significant health care economic burdens that should be reviewed according the rate of responses and should attract the attention of health policy makers and academics in many countries (9). The costs of PIs -based triple therapy for hepatitis C and adverse managements are very high per sustained viral response, which means that it is not cost effective to use these drugs in our practice at the present time.

## 1. Sofosbuvir Get Approval

Recently FDA advisory board approved the Sofosbuvir for the treatment of chronic hepatitis C patients which will open up new horizons toward noninterferon-based therapies in management of CHC patients. It is dreamed to treat these patients with one tablet daily! The FDA advisory board reviews the efficacy and safety data collected from the clinical trials and decided to register a new drug cautiously. Sofosbuvir is a nucleotide analogue inhibitor of HCV NS5B polymerase that is administered orally. It has a potent antiviral activity against all genotypes of HCV. FDA advisory board approved Sofosbuvir for treatment of naïve adults infected with genotypes 1 and 4 in combination with pegylated interferon alpha (PEG-IFN) plus ribavirin) and for treatment of naïve infected adults with genotypes 2 and 3 in combination with ribavirin which indicating that we can use the drug in protocol IFN-free therapy in genotypes 2 and 3. The drug can take orally at a single dose 400 mg daily.

The literature review showed Sofosbuvir has been studied in different populations in combination with PEG-INF and ribavirin or with other direct-acting antiviral agents for the treatment of naive patients with genotype 1 HCV infection (10-12). In ATOMIC multicenter study in naive cases with genotype 1 infection, the patients have shown good responses to the combined sofosbuvir 400 mg and PEG-IFN or ribavirin therapy within 12 weeks (12) and extension to 24 weeks did not add more virologic responses. The Sofosbuvir therapy was successful and the side effects including anemia were related to ribavirin not Sofosbuvir. Unfortunately, there is not any data about cirrhotic patients as it is difficult to treat these patients and the results of the ongoing studies will be reported soon. In contrast with the results of PIs -based triple therapy for hepatitis C, the virologic response is not depend on to the early virologic response or baseline characteristics such as IL28B CC versus non-CC genotype, high versus low baseline viral load, and genotype 1a versus genotype 1b (12). The Sofosbuvir was effective in all of these situations. One of the important findings of this study was the drug safety in responders and no resistance-associated mutations in patients with virologic failure, which is due to the high genetic barrier to HCV resistance of Sofosbuvir (12).

Numerous studies are ongoing and the preliminary reports revealed that Sofosbuvir in combination with other direct-acting antiviral agents caused significant virologic responses. In a phase 2 study on cirrhotic and null responder hepatitis C patients, the combination of sofosbuvir and simeprevir with and without ribavirin for 12 or 24 weeks in patients with HCV genotype 1 infections resulted in high (93 - 96%) SVRs (13). Perhaps in future we can use combined Simeprevir and ribavirin therapy for six months without interferon to treat different genotypes infections or Simeprevir plus ribavirin and Simeprevir for three up to six months without interferon to treat genotype 1 infections. The combination of Sofosbuvir and a second direct-acting antiviral agent such as NS5A inhibitor Ledipasvir is highly effective in treatment-naïve patients with genotype 1 HCV infection and in patients that did not respond to previous treatments (14).

## 2. Conclusion Remarks

The benefits of this new approach in eradication of HCV infection are shorter duration with safer and lower side effects reported in users in comparison with PIs -based triple therapy. Once daily oral regimen is well-tolerated without significant resistance development. Different clinical trials of Sofosbuvir on treatment-naive patients with genotypes 1 hepatitis C virus infection, through 6 months showed that patients with genotype 1 infection had higher virologic response than those underwent combination therapy and currently available triple therapies. In patients with genotypes 2 and 3 HCV infection, the efficacy was similar in both IFN-free Sofosbuvir and standard PEG-IFN/ribavirin regimens (15). Traditional predictors of treatment response, such as IL28B polymorphisms, baseline viral load, and early response did not affect the response rates.

 Opening this magic box and the emerge of Sofosbuvir reveals a hopefully future with more discovering drugs, which directly target various aspects of HCV life cycle and lead to more effective combinations of new drugs in management of HCV infection within a shorter time. Introduction of this drug will reduce to the global burden of HCV and millions of HCV infected patients around the world will be treated, and it will prevent the HCV associated morbidity and mortality such as liver cirrhosis , hepatocellular carcinoma and the need for liver transplantation , in 2020 (16). I hope we can identify the role of these drugs for the treatment of cirrhotic patients from ongoing studies. In patients with genotype 1 including relapse or resistant to therapy with low degree of liver fibrosis, we should wait for availability of new drugs.
